# Identification of a cryptic submicroscopic deletion using a combination of fluorescence in situ hybridization and array comparative genomic hybridization in a t(3;5)(q25;q35)-positive acute myeloid leukemia patient: A case report and review of the literature

**DOI:** 10.1097/MD.0000000000022789

**Published:** 2020-10-23

**Authors:** Man Gao, Shibo Li, Lina Wang, Shu Nie, Hui Pang, Xianglan Lu, Xianfu Wang, Mingwei Wang, Shirong Guo, Yuhan Ma, Fanzheng Meng

**Affiliations:** a Department of Pediatrics, the First Hospital of Jilin University, Changchun City, Jilin; b Department of Pediatrics, University of Oklahoma Health Sciences Center, Oklahoma City, Oklahoma; c Department of Radiotherapy, Public Health School of Jilin University, Changchun City, Jilin, P.R. China.

**Keywords:** acute myeloid leukemia, array comparative genomic hybridization, fluorescence in situ hybridization, submicroscopic deletion, t(3 ;5)(q25 ;q35)

## Abstract

**Rationale:**

The advent of high-resolution genome arrays including array comparative genomic hybridization (aCGH) has enabled the detection of cryptic submicroscopic deletions flanking translocation breakpoints in up to 20% of the apparently “balanced” structural chromosomal rearrangements in hematological disorders. However, reports of submicroscopic deletions flanking the breakpoints of t(3;5)(q25;q35) are rare and the clinical significance of submicroscopic deletions in t(3;5) has not been explicitly identified.

**Patient concerns:**

We present a 47-year-old man with acute myeloid leukemia. G-banding analysis identified t(3;5)(q25;q35).

**Diagnosis:**

Array CGH-based detection initially confirmed only the deletion of chromosome 3. Further characterization using fluorescence in situ hybridization identified a cryptic submicroscopic deletion including *5′ MLF1-3′ NPM1* flanking the breakpoint on the derivative chromosome 3.

**Interventions:**

The patient started “7+3” induction chemotherapy with cytosine arabinoside and daunorubicin, and subsequently received 2 cycles of high-dose intermittent acronym of cytosine arabinoside or cytarabine.

**Outcomes:**

The patient did not undergo complete remission and died from an infection due to neutropenia.

**Lessons:**

Haploinsufficiency of *NPM1* or other deleted genes, including *SSR3*, may be responsible for the phenotype of t(3;5)(q25;q35)-positive myeloid neoplasms with submicroscopic deletions.

## 1 Introduction

Following the first description of the association between t(3;5) and acute myeloid leukemia (AML) in 1976 by Rowley and Potter,^[1]^ t(3;5) is recognized as a nonrandom and uncommon abnormality occurring in AML and myelodysplastic syndromes (MDS). Although there are variable breakpoints in t(3;5), NPM1/MLF1 is the most probable fusion protein according to the literature.^[2,3]^ G-banding and fluorescence in situ hybridization (FISH) identify the majority of abnormalities, although the advent of high-resolution genome arrays, including array comparative genomic hybridization (aCGH), has enabled the detection of cryptic submicroscopic deletions flanking the translocation breakpoints in up to 20% of the apparently “balanced” structural chromosomal rearrangements in hematological disorders.^[4–7]^ However, only 3 previous reports have shown submicroscopic deletions at the breakpoint regions in *NPM1/MLF1* positive cases.^[8–10]^ Here we present the fourth report of a t(3;5)(q25;q35)-positive AML case complicated with a submicroscopic deletion on the derivative chromosome 3.

## 2 Case presentation

The medical records of a patient with t(3;5)(q25;q35)-positive AML were retrospectively reviewed. The study was approved by the Committee for Medical and Health Research Ethics of the First Hospital of Jilin University and written informed consent for publication of case details was obtained from the patient. All patient information has been anonymized.

### 2.1 Case history

A 47-year-old man originally presented to our hospital with diabetic ketoacidosis. His initial complete blood count (CBC) showed a white blood cell count (WBC) of 71.2 × 10^9^/L (no blasts were noted), a hemoglobin count of 43 g/L, and a platelet count of 21 × 10^9^/L. The peripheral blood smear showed striking dysplastic changes and rare (1–2%) blasts were seen. He had no exposure to known leukemogens and no history of chemotherapy or radiotherapy. Myeloblasts were noted at 13% in aspirate smears and at 17% in the touch preparation. Flow cytometric analysis of the aspirate identified myeloblasts at only 4%. The immunophenotype of the myeloblasts included expression of CD13 and CD33, with partial expression of CD117 and CD15, and only slight expression of CD34. The most appropriate diagnosis based on the information available at that time appeared to be refractory anemia with excess blasts-2 (RAEB-2); while acute myeloid leukemia could not entirely be excluded. He started “7+3” induction chemotherapy with cytosine arabinoside and daunorubicin. He did not completely recover, and showed residual disease after induction therapy. On the 45th day after induction therapy, biopsy showed approximately 40% blasts, consistent with persistent AML. He subsequently received 2 cycles of high-dose intermittent acronym of cytosine arabinoside or cytarabine; however, he did not completely recover and later died from an infection due to neutropenia.

### 2.2 Detection of a submicroscopic deletion in t(3;5)(q25;q35)

**Figure 1:**
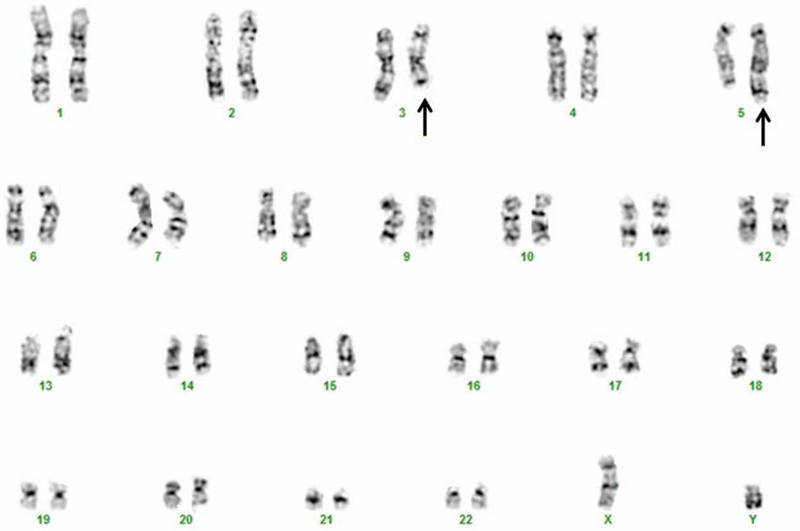
Karyotyping results. The patient's karyotype was described as 46,XY,t(3;5)(q25;q35)[13]/46,XY[7]. Arrows indicate the derivative chromosome 3 and chromosome 5.

A fresh bone marrow specimen was collected during the patient's initial visit and the specimen was analyzed cytogenetically as part of our diagnostic routine. Overnight culture was set up and harvested using Chang Marrow (Irvine Scientific, CA) according to our standard laboratory protocols.^[11]^ G-banding using trypsin-Giemsa (GTG-banding) staining was used to prepare chromosomes. Karyotypes were described according to the International System for Human Cytogenetic Nomenclature 2013 (ISCN 2013).^[12]^ Twenty metaphases were analyzed yielding the karyotype: 46,XY,t(3;5)(q25;q35)[13]/46,XY[7] (Fig. 1).

**Figure 2:**
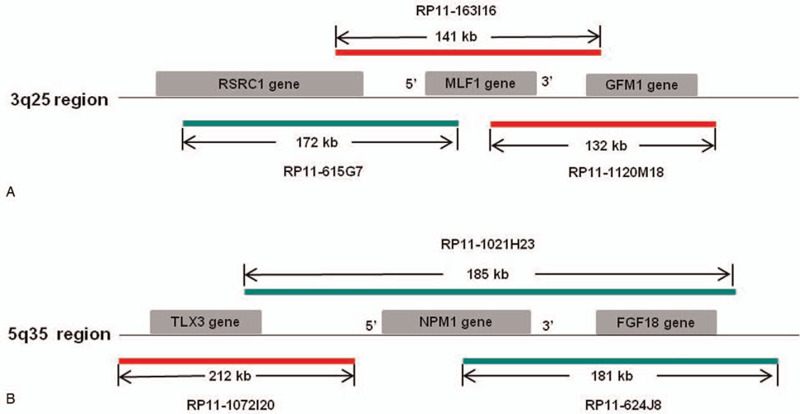
Probe schematic for the BAC clones. A. 3q25 MLF1 gene region. For the MLF1 gene, 3 BAC clones were used: RP11-615G7 was labeled with Vysis SpectrumGreen, and RP11-1120M18 and RP11-163I16 were labeled with Vysis SpectrumOrange. B. 5q35 *NPM1* gene region. For the *NPM1* gene, 3 BAC clones were used: RP11-1072I20 was labeled with Vysis SpectrumOrange and RP11-624J8 and RP11-1021H23 were labeled with Vysis SpectrumGreen. BAC = bacterial artificial chromosome.

**Figure 3:**
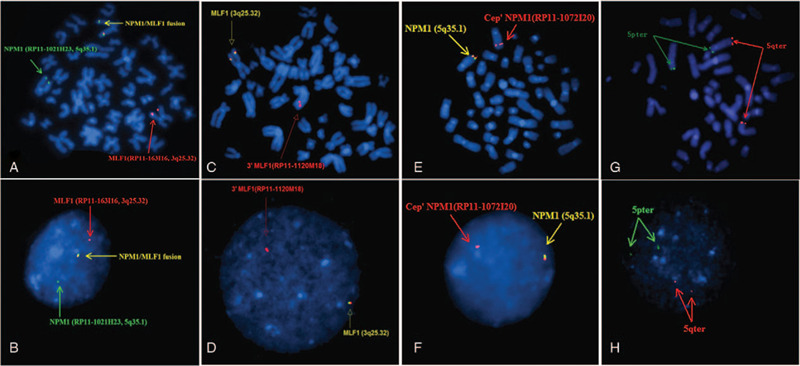
FISH results. A and B. Cohybridization with dual-color dual-fusion probes RP11-163I16 and RP11-1021H23 showed a 1R1G1F signal pattern: one *5*′ *NPM1-3*′ *MLF1* fusion signal (yellow) on the derivative chromosome 5, and 1 red signal and 1 green signal on the normal chromosome 3 and chromosome 5, respectively. There was no *5*′ *MLF1-3*′ *NPM1* yellow fusion signal on the derivative chromosome 3. C and D. FISH with dual-color break-apart probes RP11-615G7 and RP11-1120M18 showed a fusion signal (yellow) on the normal chromosome 3 and a *3*′ *MLF1* signal (red) on the derivative chromosome 5. No 5′ MLF1 green signal was detected on the derivative chromosome 3. E and F. FISH analysis with the dual-color break-apart probes hybridizing to the Cep’*NPM1* (RP11-1072I20, SpectrumOrange) and Tel’*NPM1* (RP11-624J8, SpectrumGreen) loci confirmed the loss of green signal on the derivative chromosome 3. G and H. FISH analysis using subtelomeric probes for 5pter and 5qter revealed that all cells had 2 red signals and 2 green signals. However, a red 5qter signal was present on the derivative chromosome 3, but not on the derivative chromosome 5. FISH = fluorescence in situ hybridization.

FISH analysis was performed on the fixative-preserved cell pellets obtained from the patient's bone marrow specimen taken at the initial diagnosis. All probes used are listed in Table 1 and Fig. 2 shows the probes schematically. All bacterial artificial chromosome (BAC) clones were selected according to physical and genetic mapping data of chromosome 3 and chromosome 5, as reported by the Human Genome Browser at the University of California, Santa Cruz website [http://genome.ucsc.edu/, Feb 2009 Assembly (GRCh37/hg19), last accessed April 2013] and purchased from the BAC/PAC Resources Center (Children's Hospital Oakland Research Institute, Oakland, CA). To determine whether t(3;5) formed the *NPM1*/*MLF1* fusion gene, FISH analysis was performed using cohybridization of the RP11-163I16 probe on 3q25.32 labeled with SpectrumOrange, and the RP11-1021H23 probe on 5q35.1 labeled with SpectrumGreen. Two hundred cells (including cells at metaphase and interphase) were screened. One hundred and twenty-three cells showed 1 red signal on the normal chromosome 3, 1 green signal on the normal chromosome 5, and 1 yellow (overlapped green and orange signal) fused signal on the derivative chromosome 5, resulting in a 1R1G1F signal pattern (Fig. 3A and B). The yellow fused signal represented the *5′NPM1-3′MLF1* fusion gene; however, the expected fusion signal representing the *5′ MLF1-3′ NPM1* fusion gene on the derivative chromosome 3 was absent, thereby indicating a cryptic submicroscopic deletion.

**Table 1:**
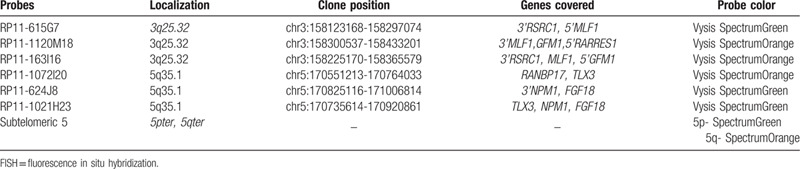
Probes used for FISH analysis.

**Figure 4:**
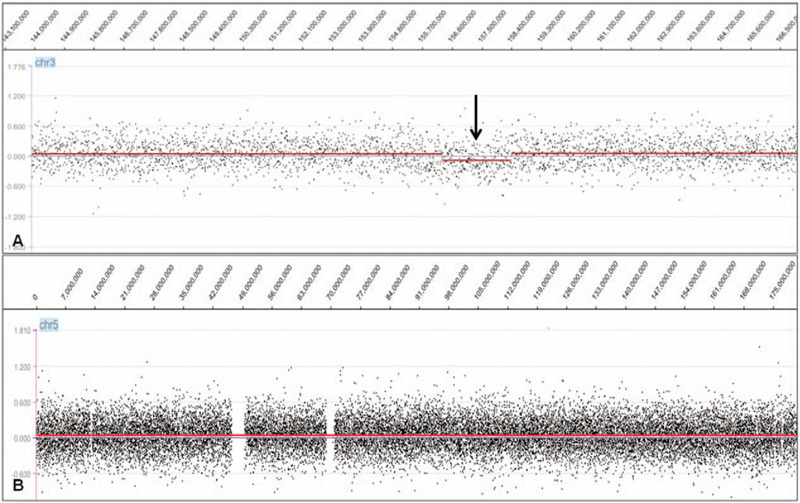
Array CGH analysis. A. Array CGH analysis of chromosome 3. A 2.016-Mb deletion from 3q25.31to 3q25.32 (156,296,873–158,312,638 on hg19) was identified for this patient, indicated by a black arrow. This deletion encompassed 10 genes: *TIPARP*, *LEKR1*, *CCNL1*, *VEPH1*, *PTX3*, *C3orf55*, *PQLC2, SHOX2*, *RSRC1,* and *5*′ *MLF1*. B. Array CGH analysis of chromosome 5. No deletion was detected on chromosome 5. CGH = comparative genomic hybridization.

Array CGH analysis was performed on genomic DNA isolated from an uncultured bone marrow specimen collected during the initial visit using a commercial DNA extraction kit (Puregene Blood Kit; Qiagen, Valencia, CA) to reveal the precise breakpoints of the microdeletion, according to the manufacturer's instructions.^[13]^ A 2.016-Mb deletion from 3q25.31 to 3q25.32 (position 156,296,873-158,312,638 on hg19) was identified in this patient (Fig. 4A). This deletion encompassed 10 genes: *TIPARP*, *LEKR1*, *CCNL1*, *VEPH1*, *PTX3*, *C3orf55*, *PQLC2, SHOX2*, *RSRC1*, and *5*′ *MLF1*. However, no deletion was detected on chromosome 5 (Fig. 4B).

Confirmatory FISH analysis using dual-color break-apart probes hybridizing to the *5*′ *MLF1* (RP11-615G7, SpectrumGreen) and *3*′*MLF1* (RP11-1120M18, SpectrumOrange) loci confirmed the loss of a *5′ MLF1* component on the derivative chromosome 3 and the presence of a *3′MLF1* component on the derivative chromosome 5 (Fig. 3C and D). Confirmatory FISH analysis using dual-color break-apart probes hybridizing to the Cep′*NPM1* (RP11-1072I20, SpectrumOrange) and Tel′*NPM1* (RP11-624J8, SpectrumGreen) loci confirmed the loss of a *3′ NPM1* and *FGF18* component on the derivative chromosome 3 (Figs. 2 and 3E, F). Meanwhile, in FISH analysis using subtelomeric probes for 5pter and 5qter (Abbott, Downers Grove, IL) all cells showed 2 red signals and 2 green signals. However, a red 5qter signal was seen on the derivative chromosome 3 but not on the derivative chromosome 5 (Fig. 3G and H). Overall, the combination of FISH and array CGH results confirmed an interstitial submicroscopic deletion including the *5*′ *MLF1-3*′ *NPM1* fusion gene on the derivative chromosome 3.

## 3 Discussion

Cryptic microdeletions surrounding the breakpoints of recurrent chromosomal translocations are well-documented, with a reported incidence ranging from 2% to 20% in various types of leukemia.^[4–6]^ Submicroscopic deletions flanking the breakpoints of t(3;5)(q25;q35) in hematologic malignancies are rarely reported. To the best of our knowledge, only 3 studies (examining 6 patients in total) have reported *5*′ *MLF1-3*′ *NPM1* deletion on the derivative chromosome 3 in myeloid malignancy with t(3;5)(q25;q35).^[8–10]^ We have described a new case in the present report. FISH tests detected a cryptic loss of the *5*′ *MLF1-3*′ *NPM1* fusion gene on the derivative chromosome 3 (Fig. 3A and B) and array CGH confirmed a microdeletion flanking the cytogenic breakpoint on chromosome 3, covering 10 genes extending from *TIPARP* to *5*′ *MLF1* (Fig. 4A). However, array CGH did not detect any deletion on chromosome 5 (Fig. 4B). Confirmatory FISH analysis using subtelomeric probes for 5pter and 5qter indicated the occurrence of t(3;5) (Fig. 3G and H). Meanwhile, FISH analysis using dual-color break-apart probes hybridizing to the Cep′*NPM1* (RP11-1072I20, orange) and Tel′*NPM1* (RP11-624J8, green) loci confirmed the loss of a *3*′ *NPM1* and *FGF18* component on the derivative chromosome 3 (Fig. 3E and F).

It has been reported that many submicroscopic deletions associated with recurrent chromosomal translocations may have an adverse prognostic impact on cancer progression.^[4,14–19]^ However, the clinical significance of the submicroscopic deletion in t(3;5) has not been explicitly identified. The *NPM1/MLF1* fusion related to t(3;5) is considered as an MDS-related abnormality by the 2016 WHO classification,^[20]^ and patients with t(3;5)(q25;q35) have a 34% survival rate after 10 years, which indicates an intermediate prognosis.^[9,21]^ Additionally, a multicenter study showed that patient characteristics of those with t(3;5) did not differ significantly from patients with normal karyotypes.^[21]^ We reviewed and analyzed the 7 reported cases with submicroscopic deletions^[8–10]^ (Table 2). All the patients were relatively young and 4 were diagnosed with AML with myelodysplasia-related changes. t(3;5) was the only cytogenetic abnormality present in all cases. The deletion of the *MLF1/NPM1* fusion gene on derivative chromosome 3 was identified using only FISH analysis in 5 cases; in another 2 cases, including the present case, the fusion gene was detected using a combination of FISH and a high resolution technique (single nucleotide polymorphism or CGH array). Unfortunately, for the former 5 cases there was no information regarding prognoses, so we could not predict the possible impact of these submicroscopic deletions on prognosis. In the present case, the patient did not achieve complete remission after induction therapy and died 4 months after the diagnosis, although 1 previously reported case^[10]^ showed a relatively favorable prognosis. We analyzed the gene content of deleted region to investigate the possible involvement of specific genes in the clinical phenotype.^[10]^ The genes *TIPARP*, *LEKR1*, *CCNL1*, *VEPH1*, *PTX3*, *C3orf55*, *PQLC2*, *SHOX2*, *RSRC1*, and *5*′ *MLF1* from chromosome 3, and *3*′ *NPM1* and *FGF18* from chromosome 5 were deleted in both cases. *KCNAB1* and *SSR3* from chromosome 3, and *FBXW11*, *STK10*, *EFCAB9*, *UBTD2*, and *SH3PXD2B* from chromosome 5 were not deleted in the present case. Of all the undeleted genes, *SSR3* (a member of signal sequence receptor family) is heavily involved in cell growth and differentiation and closely associated with many tumor types. *SSR3* acts as a novel oncogene in hepatocellular carcinoma (HCC) and therefore can serve as a biomarker for the prognosis of HCC patients.^[22]^ However, there are no reports of a relationship between this gene and hematological disorders. Further experiments are needed to confirm whether *SSR3* is related with the poor prognosis of the present case. Additionally, it has been reported that *NPM1* haploinsufficiency acts together with the 5′*NPM1*-3′*MLF1* fusion gene to enhance myeloid progenitor activity^[23]^; thus, we cannot rule out the possibility that haploinsufficiency of *3*′ *NPM1,* or of other deleted genes in our case, may adversely affect disease progression. However, we cannot draw conclusions from this single case.

**Table 2:**
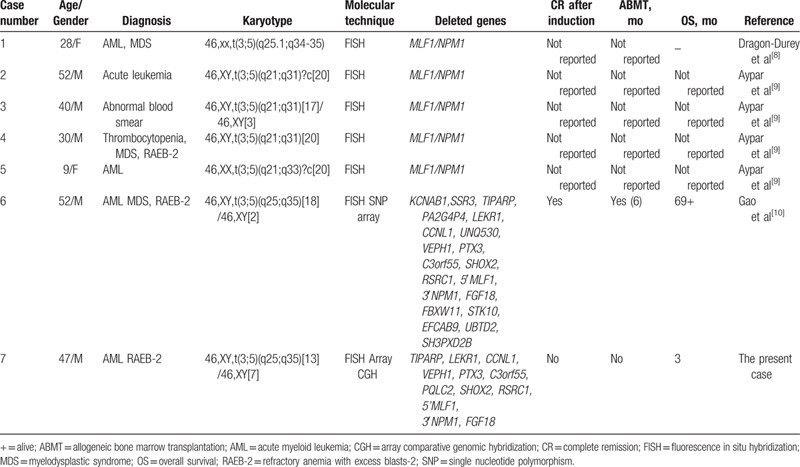
Clinico-biological characteristics of the *NPM1*/*MLF1* positive cases with submicroscopic deletion.

As we know, array CGH provides an accurate, cost-effective, and time-efficient whole-genome analysis at a significantly higher resolution than that of conventional karyotyping and FISH.^[24–27]^ However, in the present case, array CGH detected the cryptic microdeletion flanking the cytogenetic breakpoint on chromosome 3, while it did not detect any deletion on chromosome 5. Additional FISH analyses confirmed the loss of a *3*′ *NPM1* and *FGF18* component on the derivative chromosome 3. According to the literature,^[24,28,29]^ array CGH has advantages in identification of cryptic imbalances and detection of clonal aberrations in non-dividing cancer cells or samples with a resolution of at least 0.1 Mb, but it cannot detect balanced rearrangements or genomic imbalances that are present in <10% to 20% of the cells.^[30–32]^ FISH can also detect genomic abnormalities in metaphase or non-dividing interphase cells with a resolution of 150 to 900 kb, depending on the probe size, and it may detect as low as 3% to 10% of the abnormal cells.^[24,26,33]^ In the present case, the percentage of abnormal cells in cultured bone marrow was confirmed as 61.5% through FISH analysis, but it was impossible to determine in uncultured bone marrow because of sample deficiencies. Additionally, we examined Nimblegen's chips and found that only 2 probes on the chips cover the chr5:170825116-171006814 (RP11-624J8) region. Maybe our array could not detect this deletion because of the lower percentage of abnormal cells or the lower density of oligonucleotide probes in the deleted region. Nevertheless, in contrast to FISH, microarray analysis utilizes data from multiple oligonucleotide probes and does not require pre-existing knowledge of possible regions of interest, adding power to reveal low level mosaicism and cryptic alterations throughout the entire genome.^[25–27,30]^ The combination of FISH and array CGH can therefore better identify cryptic aberrations.

## 4 Conclusion

In summary, we identified a cryptic submicroscopic deletion flanking the breakpoint in a t(3;5)(q25;q35)-positive AML case using a combination of FISH and array CGH. Even though the loss of genes flanking the breakpoints in t(3;5)(q25;q35) may possibly impact disease prognosis, solid conclusions cannot be drawn due to the limited number of cases. Through comparisons with previously reported cases, we analyzed the function of related genes and deduced that haploinsufficiency of *NPM1* or other deleted genes, including *SSR3,* may be responsible for the phenotype of the t(3;5)(q25;q35) myeloid neoplasms. Additionally, the abnormal cell percentage and the density of oligonucleotide probes may affect array CGH results; thus, the combination of FISH and array CGH can better identify cryptic aberrations and shed light on clinical anomalies.

## Author contributions

**Conceptualization:** Man Gao, Fanzheng Meng, Shibo Li.

**Data curation:** Mingwei Wang, Shirong Guo, Yuhan Ma.

**Investigation:** Man Gao, Shu Nie, Fanzheng Meng.

**Methodology:** Hui Pang, Xianglan Lu, Xianfu Wang, Shibo Li.

**Supervision:** Shibo Li.

**Writing – original draft:** Man Gao.

**Writing – review & editing:** Fanzheng Meng, Shibo Li.
